# Assessing environmental factors associated with regional schistosomiasis prevalence in Anhui Province, Peoples’ Republic of China using a geographical detector method

**DOI:** 10.1186/s40249-017-0299-x

**Published:** 2017-04-17

**Authors:** Yi Hu, Congcong Xia, Shizhu Li, Michael P. Ward, Can Luo, Fenghua Gao, Qizhi Wang, Shiqing Zhang, Zhijie Zhang

**Affiliations:** 1 Department of Epidemiology, School of Public Health, Fudan University, Shanghai, 200032 China; 2 Key Laboratory of Public Health Safety, Ministry of Education, Shanghai, China; 3 Laboratory for Spatial Analysis and Modeling, School of Public Health, Fudan University, Shanghai, China; 4 Collaborative Innovation Center of Social Risks Governance in Health, School of Public Health, Fudan University, Shanghai, China; 5 National Institute of Parasitic Diseases, Chinese Center for Disease Control and Prevention, Key Laboratory of Parasite and Vector Biology, Ministry of Health; WHO Collaborating Center for Tropical diseases, Shanghai, People’s Republic of China; 6 Faculty of Veterinary Science, The University of Sydney NSW, Sydney, Australia; 7 Department of Environmental Art and Architecture, Changsha Environmental Protection Vocational Technical College, Changsha Hunan, People’s Republic of China; 8 Anhui Institute of Parasitic Diseases, Wuhu, People’s Republic of China; 9 No.130 Dong’an Road, Xuhui District, Shanghai, 200032 China

**Keywords:** *Schistosoma japonicum*, Geographical detector, Spatial variation analysis, Environmental factors, Geographic information systems, China

## Abstract

**Background:**

Schistosomiasis is a water-borne disease caused by trematode worms belonging to genus *Schistosoma*, which is prevalent most of the developing world. Transmission of the disease is usually associated with multiple biological characteristics and social factors but also factors can play a role. Few studies have assessed the exact and interactive influence of each factor promoting schistosomiasis transmission.

**Methods:**

We used a series of different detectors (i.e., specific detector, risk detector, ecological detector and interaction detector) to evaluate separate and interactive effects of the environmental factors on schistosomiasis prevalence. Specifically, (i) specific detector quantifies the impact of a risk factor on an observed spatial disease pattern, which were ranked statistically by a value of Power of Determinate (*PD*) calculation; (ii) risk detector detects high risk areas of a disease on the condition that the study area is stratified by a potential risk factor; (iii) ecological detector explores whether a risk factor is more significant than another in controlling the spatial pattern of a disease; (iv) interaction detector probes whether two risk factors when taken together weaken or enhance one another, or whether they are independent in developing a disease. Infection data of schistosomiasis based on conventional surveys were obtained at the county level from the health authorities in Anhui Province, China and used in combination with information from Chinese weather stations and internationally available environmental data.

**Results:**

The specific detector identified various factors of potential importance as follows: Proximity to Yangtze River (0.322) > Land cover (0.285) > sunshine hours (0.256) > population density (0.109) > altitude (0.090) > the normalized different vegetation index (NDVI) (0.077) > land surface temperature at daytime (LST_day_) (0.007). The risk detector indicated that areas of schistosomiasis high risk were located within a buffer distance of 50 km from Yangtze River. The ecological detector disclosed that the factors investigated have significantly different effects. The interaction detector revealed that interaction between the factors enhanced their main effects in most cases.

**Conclusion:**

Proximity to Yangtze River had the strongest effect on schistosomiasis prevalence followed by land cover and sunshine hours, while the remaining factors had only weak influence. Interaction between factors played an even more important role in influencing schistosomiasis prevalence than each factor on its own. High risk regions influenced by strong interactions need to be targeted for disease control intervention.

**Electronic supplementary material:**

The online version of this article (doi:10.1186/s40249-017-0299-x) contains supplementary material, which is available to authorized users.

## Multilingual abstracts

Please see Additional file 1 for translations of the abstract into six working languages of the United Nations.

## Background

Schistosomiasis, caused by a trematode worms belonging to the genus *Schistosoma* [1], is a chronic, debilitating disease that occurs in tropical and subtropical environments, where it remains a burden of major public health and economic significance [2]. An estimated 779 million people live in schistosome-endemic areas with more than 200 million individuals currently infected [3]. The global burden of schistosomiasis has been estimated at 3.3 million disability-adjusted life years (DALYs) according to the latest estimate of the global burden of diseases (GBDs) [4], but the true burden could be considerably greater than previously expected [5].

Transmission of schistosomiasis is usually associated with multiple biological characteristics and social factors, which influence vector biology, ecology, economic and policy factors [6]. For example, climatic and environmental conditions suitable for both parasite and intermediate host snail, coupled with inadequate water supply at home, sanitation and poor hygiene conditions, are the root causes for the persistence of schistosomiasis prevalence [7]. Understanding the relationship between risk factors and schistosomiasis is of great importance as it supports the implementation of effective control programs.

It is also important to note that most of the preceding work on schistosomiasis is based on analyzing prevalence data, employing conventional statistical approaches [8, 9] or Bayesian spatial statistics [10–12]. However, these models usually assume that the response variable (e.g., occurrence of schistosomiasis infection) follows a certain statistical distribution (e.g., binomial) and violation of such assumptions, which is often the case in practice (e.g., when the sample sizes are small), can have a major impact on model validity. Besides, problems can occur when dealing with a nominal covariate that has many categories with multiple regression models [13]. To add such nominal covariates to the model effectively adds “noise” or unreliability and thus poses a difficulty in model building. Furthermore, it is difficult to interpret interactive effects of covariates in classic models and the inclusion of interactions when a study is not specifically designed to assess them can make it difficult to estimate the other effects on the model [14]. Therefore, there is a need to develop better on more suitable techniques for assessing the association between health outcome and risk factors.

In this study focused on schistosomiasis, we used a method of a series of detectors based on variables commonly used in geographical information systems (GIS) as proposed by Wang et al. [15] to assess risk factors associated with health outcomes by means of spatial variance analysis (SVA). The basic idea of SVA is to measure the degree according to which the spatial distribution of the health outcome (e.g. schistosomiasis prevalence) is consistent with that of the risk factors. Based on this idea, four geographical detectors (specific detector, risk detector, ecological detector and interaction detector) were used to assess the potential association with the health outcome, i.e. prevalence of schistosomiasis. We first mapped the spatial distribution of schistosomiasis prevalence in Anhui Province at the county level and then evaluated the potential influence of the risk factors. Finally, we employed the four detectors to assess the association between prevalence and these factors.

## Methods

### Approach and study area

Techniques of geographic information system (GIS), satellite-generated remote sensing (RS) and the geographical detector approach were combined for integrated risk modelling of *Schistosoma japonicum*. The analysis was conducted at the county level, and the geographical focus was Anhui Province in eastern China.

### Parasitological data

**Fig. 1:**
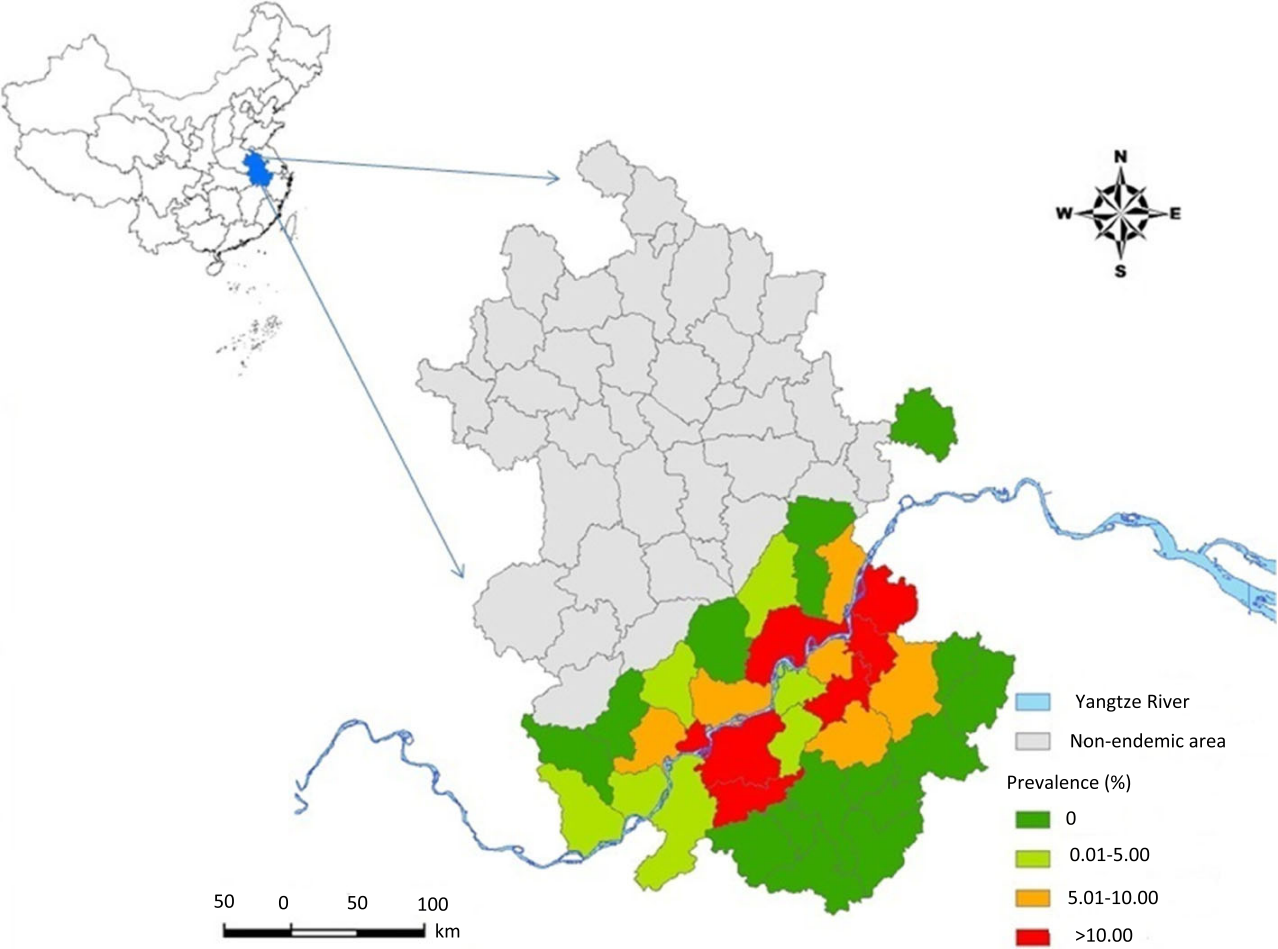
Prevalence of schistosomiasis at the county level in Anhui Province, China

The *S. japonicum* infection prevalence data were collected from a cross-sectional, survey carried out by health professionals of the Anhui Institute of Parasitic Diseases in November 2005. The databases in China are county-based, with all reported schistosomiasis cases and the population at risk given at the county level. These data were originally collected through village-based field surveys using a two-pronged diagnostic approach [screening by a serological test on all residents of 5 to 65 years old followed by confirmation by a faecal parasitological test (Kato-Katz technique)] [16] for those with positive serology. The data were collated at the township level and the reported data were summed at the county level. At the time of the collection of the study data, there were 39 schistosome-endemic counties and 39 non-endemic counties in Anhui Province. A map of prevalence of schistosomiasis at the county level is shown in Fig. 1.

### Environmental data

The environmental data utilized for the study can be grouped as follows:

(i) *Physical factors* These included the land surface temperature (LST), the normalized different vegetation index (NDVI), sunshine hours and altitude. LST and NDVI were derived from the Level 1 of the Atmosphere Archive and Distribution System (https://earthdata.nasa.gov/about/daacs/daac-laads). Eight-day composite images with 1-km resolution for the year 2005 were downloaded from the website. These images were georeferenced and sub-set in ERDAS 2011 software (https://www.gim-international.com/content/news/erdas-2011-software). ArcGIS, version 10.0 software (ESRI; Redlands, CA, USA) was used to extract average LST and NDVI data for each pixel of the image. Monthly sunshine hours in 2005 were derived from the China Meteorological Data Sharing Service System (http://www.cma.gov.cn/2011qxfw/2011qsjgx/). With available data from 756 meteorological stations, Kriging interpolation was used to derive continuous overlays of sunshine hours for each month. The average values for each pixel of these overlays were also extracted within ArcGIS 10. Altitude data were obtained from the digital elevation model (DEM) from the Shuttle Radar Topography Mission (SRTM), an international project spearheaded by the U.S. National Geospatial-Intelligence Agency (NGA) and the U.S. National Aeronautics and Space Administration (NASA).
(ii) *Social factors* These included the distance to Yangtze River, land cover and population density. The shape file data of Yangtze River were downloaded from Conservation Science Data Sets of World Wild Foundation at http://worldwildlife.org. Proximity to Yangtze River was regarded as a social factor given the fact that it reflects local activities, i.e. the closer to the river, the higher the chance to get infected. To assess the effect of proximity to Yangtze River, buffers around the Yangtze River were drawn using ArcGIS 10.0. Land cover data for Anhui in 2004 were obtained from China’s Ministry of Land and Resources (MLR). It includes six major types (cultivated land, forest, grass land, water body, unused land and rural/urban settlements) and 25 sub-categories. Considering the environment suitability with respect to breeding of freshwater snails, we reclassified the land cover factor into: paddy fields, dry land, forest, grass land, water body and other (which included used land and rural/urban settlement). Population density data were sourced from Center for International Earth Science Information Network (CIESIN) at Columbia University, USA (http://sedac.ciesin.columbia.edu/data/sets/browse).

### Statistical analysis

**Fig. 2:**
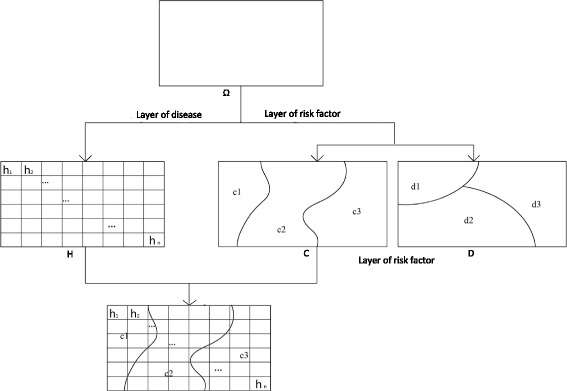
Layers of schistosomiasis (H) and risk factors (C and D). H is measured by the prevalence in grids and C and D are stratified by their attributes

The main idea of the geographical detector system used here is that if a risk factor dominates a disease, then the spatial distribution of the factor is consistent with that of the disease. The mechanism is quantified by power values as follows:

In the study area Ω, let schistosomiasis be measured by prevalence in grids, h_1_, h_2_,…, h_n_ and let C and D be two potential risk factors associated with the infection (as shown in Fig. 2). Measurements of C and D can be the continuous or categorical variable, then Ω is assumed to be stratified by the attribute of C and D (which are usually fixed) and denoted as subareas {c1, c2, c3} and {d1, d2, d3}, respectively. The schistosomiasis layer H is overlaid by a potential factor layer, such as D. The average prevalence (or morbidity rate), together with their variances of schistosomiasis prevalence in each subarea and in the whole study area Ω, are denoted by ${\overline{y}}_{d1}$, ${\overline{y}}_{d2}$, ${\overline{y}}_{d3}$, ${\overline{y}}_D$ and Vard_1_, Vard_2_, Vard_3_, VarD, respectively. If schistosomiasis prevalence is completely dominated by factor D, the prevalence (or morbidity rate) in grids *h*_*i*_ will be homogeneous in each of the subareas {d_1_, d_2_, d_3_} and therefore, Vard_i_ (i = 1, 2, 3) will be zero; if schistosomiasis prevalence is completely independent of factor D, the accumulated area’s weighted dispersion variances of the prevalence in the subareas will be no different from the pooled area’s weighted dispersion variances of the study area Ω. The mechanism is measured by the Power of Determinant (*PD*):

1 $$ P D=1-\frac{\left({N}_{d1} Va{r}_{d1}+{N}_{d2} Va{r}_{d2}+{N}_{d3} Va{r}_{d3}\right)}{N\times Va{r}_D} $$

where N and N_di_ denote the areas of the study area Ω and the subarea d_i_, respectively. The *PD* value actually explains how much variation of the prevalence can be controlled by the distribution of the risk factor. If factor D completely controls schistosomiasis, *PD* equals 1; if it is completely unrelated to schistosomiasis, *PD* equals 0. The value of *PD* lies in [0, 1]. The larger the value of *PD*, the greater the impact of factor D on schistosomiasis prevalence. The *PD* value, therefore, can be used to quantify the association between schistosomiasis prevalence and the risk factors studied.

Specifically, the geographical detectors, based on *PD*, are composed of the following four detectors:

(i) specific detector It quantifies the impact of a risk factor on an observed spatial disease pattern;
(ii) risk detector It detects high risk areas of a disease on the condition that the study area is stratified by a potential risk factor;
(iii) ecological detector It explores whether a risk factor is more significant than another in controlling the spatial pattern of a disease;
(iv) interaction detector It probes whether two risk factors when taken together weaken or enhance one another, or whether they are independent in developing a disease.

A detailed discussion about the four detectors can be seen in the Appendix.

The density of grid h_i_ can be specified based on the research objective. The more grid points there are, the higher the resulting accuracy, but also the greater the time consumed and therefore a balance is needed in practice. We set grid h_i_ to be 1 × 1 km in line with the spatial resolution of RS data on climatic conditions. The software of geographical detector used in the study can be freely downloaded at http://www.sssampling.org/Excel-GeoDetector.

## Results

The specific detector identified the significant risk factors and their relative influence on schistosomiasis prevalence ranked by *PD* value as follows (Table 1): proximity to Yangtze River (0.322) > Land cover (0.285) > sunshine hours (0.256) > population density (0.109) > DEM (0.090) > NDVI (0.077) > LST_day_ (0.007).

**Table 1:** Values of Power of Determinate (PD) for risk factors

Factors	Proximity to Yangtze	Land cover	Sunshine hours	Population density	DEM	NDVI	LST_day_
PD value	0.322	0.285	0.256	0.109	0.090	0.077	0.007

The ecological detector (Table 2) showed that the difference of *PD* between proximity to Yangtze River, land cover, and sunshine hours were not statistically significant; the differences between the remaining factors were not statistically significant either; however, the differences between any one of the first three factors and any one of the remaining factors were statistically significant. Results of the specific detector and the ecological detector suggested that proximity to Yangtze River, land cover, and sunshine hours can be classified into important factors that had strong effect on schistosomiasis prevalence, while the remaining factors can be grouped into factors of weak influence.

**Table 2:** Statistically significant differences of the influence of risk factors on schistosomiasis

Factors	Proximity to Yangtze	Land cover	Sunshine hours	Population density	DEM	NDVI	LST_day_
Proximity to Yangtze							
Land cover	N^b^						
Sunshine hours	N^b^	N^b^					
Population density	Y^a^	Y^a^	N^b^				
DEM	Y^a^	Y^a^	N^b^	N^b^			
NDVI	Y^a^	Y^a^	N^b^	N^b^	N^b^		
LST_day_	Y^a^	Y^a^	N^b^	N^b^	N^b^	N^b^	

^a^the difference of influence between the two factors is significant at the 95% confidence level

^b^the difference of influence between the two factors was not significant at the 95% confidence level

The risk detector uncovered that the average prevalence rates of schistosomiasis prevalence in each buffer region of Yangtze River were 3.89‰ (0–10 km), 2.87‰ (10–50 km), 0.83‰ (50–100 km) and 0.01‰ (>100 km), respectively. Table 3 shows that there was a significant difference in the average prevalence between each buffer zone. Note that the average prevalence decreased dramatically from the 10–50 km buffer to that of 50–100 km emphasizing the strong influence on risk by Yangtze River. Risk analysis with respect to the prevailing land cover is presented in Table 4, which shows that the average prevalence of schistosomiasis is the highest in the grass lands (3.44‰), which is significantly different from that of the other types of land cover.

**Table 3:** Statistically significant differences of the average prevalence between four distance buffers of Yangtze River

Buffer	0-10 km	10-50 km	50-100 km	>100 km
0-10 km				
10-50 km	Y^a^			
50-100 km	Y^a^	Y^a^		
>100 km	Y^a^	Y^a^	Y^a^	

^a^the difference of influence between the two factors is significant at the 95% confidence level

**Table 4:** Statistically significant differences between the average prevalence rates between six types of land cover

Land cover (average prevalence)	Paddy fields	Dry land	Forest	Grass land	Water body	Other
Paddy fields (1.77‰)						
Dry land (2.41‰)	Y^a^					
Forest (1.58‰)	Y^a^	Y^a^				
Grass land (3.44‰)	Y^a^	Y^a^	Y^a^			
Water body (1.98‰)	N^b^	N^b^	Y^a^	Y^a^		
Other (2.07‰)	N^b^	N^b^	Y^a^	Y^a^	N^b^	

^a^the difference of influence between the two factors is significant at the 95% confidence level

^b^the difference of influence between the two factors was not significant at the 95% confidence level

Table 5 shows the mutual interaction between the seven factors investigated arranged so the strength provided by each pair of factors can be seen. The interactive effect between proximity to Yangtze River and land cover was found to enhance each other (Yangtze River ∩ sunshine hours (0.388) > max (Yangtze River (0.322), land cover (0.285))) to increase the schistosomiasis prevalence, whereas the interactions between proximity to Yangtze River and LST_day_ was found to unilaterally weaken the influence of Yangtze River to decrease the schistosomiasis prevalence (min (Yangtze River (0.322), LST (0.007)) < Yangtze River ∩ LST (0.306) < max (Yangtze River (0.322), LST (0.007))). Note that the interactions between the most important factors (proximity to Yangzte River, land cover, and sunshine hours) mutually enhance their separate impacts.

**Table 5:** Interactions (measured by PD value) between pairs of risk factors

Factors	Proximity to Yangtze	Land cover	Sunshine hours	Population density	DEM	NDVI	LST_day_
Proximity to Yangtze							
Land cover	0.388						
Sunshine hours	0.372	0.333					
Population density	0.365	0.201	0.205				
DEM	0.349	0.184	0.198	0.104			
NDVI	0.331	0.121	0.151	0.148	0.080		
LST_day_	0.306	0.099	0.040	0.110	0.040	0.035	

## Discussion

In this study, we used four geographical detectors to assess effects of environmental factors on schistosomiasis prevalence. We believe this method to be “not classic” in that it offers a new approach to extracting the implicit interrelationships between a health outcome and risk factors without any assumptions or restrictions with respect to the response variable, and it detects the spatial patterns of risk factors and health outcome which are difficult to model using classic epidemiological methods. Perhaps most importantly, it quantifies interactive effects between factors which are difficult to estimate and interpret in classic models. Geographical detectors have been successfully used to explore determinants and their interaction with tube defects [15], the under-five mortality in earthquake [17], typhoid and paratyphoid fever [18], typhoid cancer [19], hand-foot-mouth disease [20], and Class B notifiable disease [21]. Over the past decades, there has been increasing attention to schistosomiasis-related factors, and the challenges that their complex interactions present to public health services and control programs [5]. This paper demonstrates how the detector system used here was used to provide some clues to these issues.

**Fig. 3:**
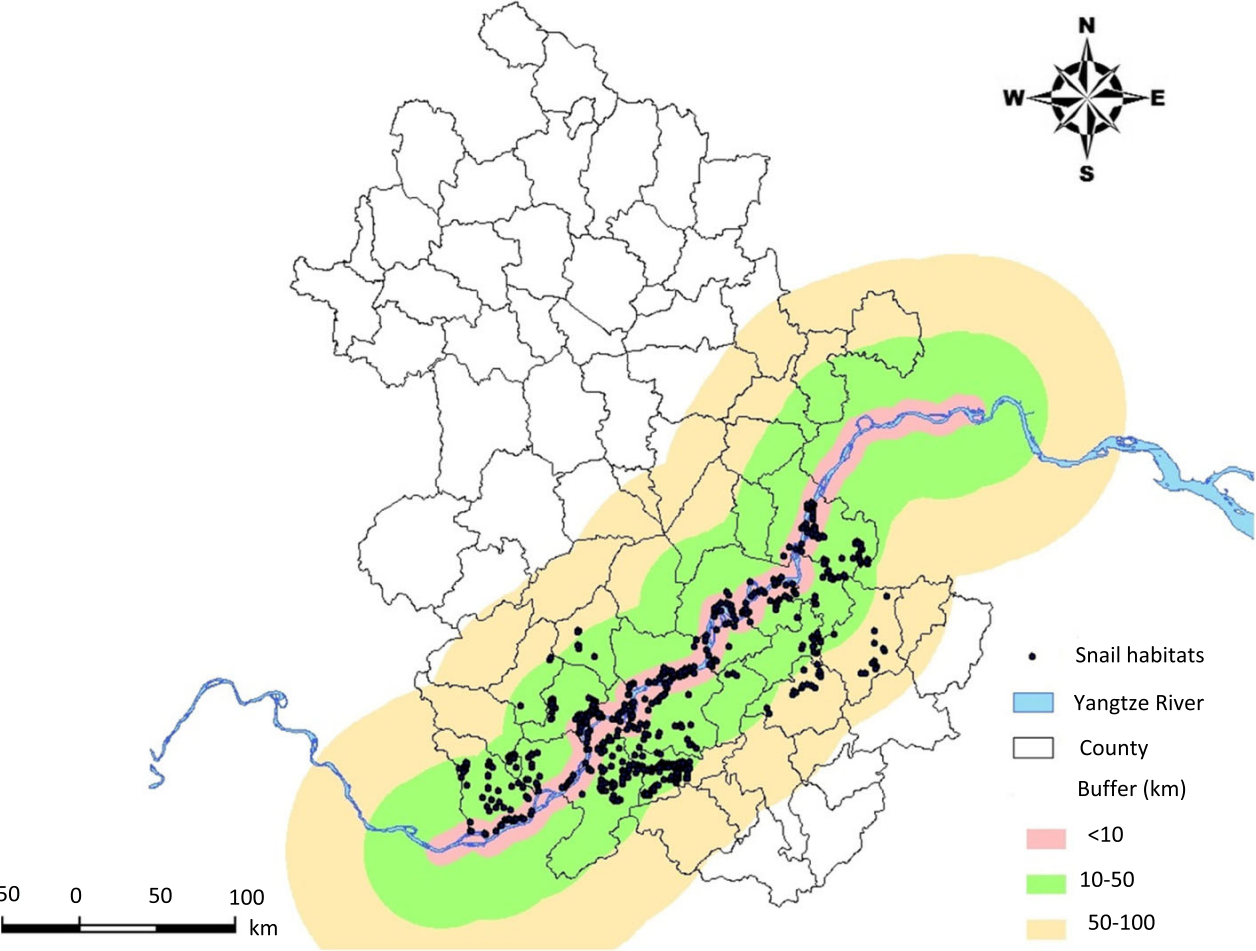
Locations of snail habitats in Anhui Province, China. Three buffer regions of the Yangtze River were overlaid

With the four geographical detectors, we found that proximity to Yangtze River had the strongest effect on schistosomiasis prevalence followed by land cover and sunshine hours, while the remaining factors had only weak influence. The observed risk factors found to be related to *S. japonicum* infection are well interpretable with the epidemiology of schistosomiasis and known biology of snails. Studies confirm that the snail habitats of are widely distributed in the lower reaches of Yangtze River [22]. Frequent flooding, which is common, snails in these habitats can be dispersed and deposited widely in various other localities, such as rivers, lakes, and wetland. Hence, risky water contact is more likely for individuals living on or near the shore and engaging in agricultural activities and fishing. The buffer regions of Yangtze River can thus be regarded as indicators of exposure. In our study area, snail habitats were mainly located within a buffer distance of 50 km from the Yangtze River (Fig. 3). This also explains why schistosomiasis prevalence decreased significantly in the regions beyond the 10–50 km buffer. The risk detector disclosed that the grass land is the highest risk (average prevalence of 3.44‰) among other types of land cover, which is because grass land provides ideal breeding habitats for snails. Climate conditions, such as daylight and LST, have been shown to influence the distribution and density of snails and the rate of schistosomal development in the snail host [23–25]. Our study, however, shows that only sunshine hours was responsible for the spatial pattern of schistosomiasis prevalence, while LST had week influence.

Of more interest is how interactions between environmental factors influence schistosomiasis prevalence. Heavily schistosomiasis-affected areas are usually influenced by a mixed interaction of multiple factors [3]. It is difficult to assess and interpret interactions using classic epidemiological methods if there are too many risk factors, while the interaction detector used here explores the interactive effect by overlaying spatial pattern of risk factors and quantifies it using the *PD* value. This makes it possible, and easy, to interpret and compare interactions with separate effects. Our analysis shows that interactions between proximity to Yangtze River and other environmental factors had (unilaterally) enhanced the separate effect of proximity to Yangtze River (except LST_day_) and that interactions between the weakly influencing factors (unilaterally or nonlinearly) enhanced their single effects as well. These findings suggest that interactions between risk factors play an important role in influencing schistosomiasis prevalence and should be accounted for when planning control interventions.

The risk detector can identify high risk regions so that priority prevention and disease intervention can be taken. Our results uncover that the buffer region of 10 km around Yangtze River and region of grass land were of great concern. In particular, the region intersected by the above two regions should be given a priority, as this small area is an accurate location of high risk. A specific intervention here would be particularly efficient and thus streamline the use of limited resources.

The present study highlights some limitations that should be noted. First, the geographical detector approach is based on spatial variance analysis of the spatial consistency of health risk distribution with suspected risk factors. If the risk factors do not present spatial patterns (e.g., patients’ age and gender) or the study area is too small to display a spatial pattern, it is difficult to identify these factors without a field sampling survey for suspect factors [15]. Second, it is somewhat subjective to deal with quantitative factors compared to qualitative factors, the values of which are determined by their nature or attributes (e.g., land cover) because arbitrary methods of discretization (e.g., equal interval and quantile) may not characterize actual association between risk factors and a health outcome. Therefore, some prior knowledge would be helpful in discretizing quantitative variables. Finally, uncertainty about the *PD* value has not been considered yet and this constitutes an area for further work.

In general, the causes of many diseases are complicated and health resources are limited in undeveloped areas. Therefore, tools, such as the geographical detector system presented here are extremely welcome that are relatively easy and efficient to implement in determinant detection for priority prevention and disease intervention. These detectors, we believe, can be used for other environment-related diseases where there are complex relationships between exposure and the health outcome of interest.

## Conclusions

This study presents an application of a series of geographical detectors in assessing environmental factors associated with schistosomiasis prevalence in Anhui Province. It was found that proximity to Yangtze River, land cover, and sunshine hours were the main factors responsible for schistosomiasis prevalence and that most interactions between risk factors enhanced their single effects.

## Additional file

Additional file 1: Multilingual abstracts in the six official working languages of the United Nations. (PDF 411 kb)

## Abbreviations

CIESIN: Center for International Earth Science Information Network (CIESIN)
DALYs: Disability-adjusted life years
DEM: Digital elevation model
GBDs: The global burden of diseases
GIS: Geographical information systems
LST: Land surface temperature
MLR: Ministry of Land and Resources
NDVI: Normalized difference vegetation index
PD: Power of determinate
RS: Remote sensing
SRTM: The Shuttle Radar Topography Mission
SVA: Spatial variance analysis
