# Supplementary material for: Assessing environmental factors associated with regional schistosomiasis prevalence in Anhui Province, Peoples’ Republic of China using a geographical detector method
Source: Infect Dis Poverty. 2017 Apr 17;6:87. doi: 10.1186/s40249-017-0299-x (PMC5392949; doi:10.1186/s40249-017-0299-x)
Supplement: Additional file 1: — Multilingual abstracts in the six official working languages of the United Nations. (PDF 411 kb) [file 40249_2017_299_MOESM1_ESM.pdf]

Translation of the abstract into the six official working languages of the United Nations

تقييم العوامل البيئية المرتبطة بانتشار مرض البلهارسيا محليا في مقاطعة آنهوي بجمهورية الصين الشعبية باستخدام أسلوب الكشف الجغرافي

يي هوجين، كونجكونج شيا، شيجو لي، مايكل ب. وارد، كان لوه، فينغواه جاو، تشي وانغ، شيقينج تشانغ، جيجي تشانغ

الملخص

الخلفية: البلهارسيا هي مرض ينتقل عن طريق المياه وتنتج عن الإصابة بالديدان التريماتودية من جنس البلهارسيا وتعتبر أكثر انتشارا في العالم النامي ويرتبط إنتقال المرض عادة بالعديد من العوامل البيولوجية والاجتماعية التي تلعب دورا هاما في الانتقال ولكن هناك عدد قليل من الدراسات التي قيمت التأثير الدقيق لكل عامل من هذه العوامل على انتقال البلهارسيا.

الأساليب: تم إستخدام سلسلة من الكواشف المختلفة (كاشف نوعي، كاشف خطورة، كاشف بيئي، وكاشف تداخلي) وذلك لتقييم التأثير الفردي والمتداخل للعوامل البيئية علي إنتشار البلهارسيا، على وجه التحديد، (i) الكاشف النوعي يحدد تأثير عامل الخطورة علي نمط المرض المكاني الملاحظ، والذي تم تصنيفه إحصائيا تبعا لقيمة حساب القوة التعويضية (PD)، (ii) كاشف الخطورة يكشف مناطق المرض عالية الخطورة في منطقة الدراسة مقسمة تبعا لعامل خطورة محتمل، (iii) كاشف بيئي يوضح إذا ما كان كاشف الخطورة أكثر أهمية من الكواشف الأخرى في السيطرة علي النمط المكاني للمرض، (iv) الكاشف التداخلي يكشف عن ما إذا كان أخذ اثنين من عوامل الخطورة يضعف أو يعزز كل منهما الأخر او إذا كان كل منهما مستقل كسبب في الإصابة بالمرض، تم جمع بيانات الإصابة بالبلهارسيا استناداً إلى الدراسات الاستقصائية التقليدية التي تم الحصول عليها على مستوى المحافظة من السلطات الصحية في مقاطعة آنهوي بالصين وتم إستخدامها مع المعلومات التي تم الحصول عليها من محطات الأرصاد الجوية الصينية والبيانات البيئية المتاحة على الصعيد الدولي.

النتائج: الكاشف النوعي تعرف على العديد من العوامل ذات الأهمية المحتملة علي النحو التالي: القرب من نهر اليانغتسى (0.322)> الغطاء الأرضي (0.285)> ساعات سطوع الشمس (0.256) > الكثافة السكانية (0.109) > الارتفاع عن سطح الأرض (0.090) > مؤشر إختلاف الغطاء النباتي (0.077) > درجة حرارة سطح الأرض خلال النهار (0.007) وأشار كاشف الخطورة إلي ان أن مجالات البلهارسيا عالية المخاطر تقع على مسافة 50 كم من نهر اليانغتسي وبين المؤشر البيئي ان العوامل التي تم دراستها كان لها تأثيرا مختلفا كبيرا كما أوضح كاشف التداخل أن التفاعل بين العوامل عزز من تأثيرها الرئيسي في معظم حالات إنتشار المرض.

الخلاصة: كان للقرب من نهر اليانغتسى أثرا قويا على انتشار مرض البلهارسيا وتبعه في التأثير كلا من الغطاء الأرضي وساعات سطوع الشمس بينما كان لباقي العوامل أثرا ضعيفا ولعب التداخل بين العوالم المختلفة دورا أكثر أهمية في إنتشار البلهارسيا من دورها منفردا لذلك فإن المناطق التي تتأثر بالتداخلات القوية بين العوامل محل الدراسة يجب إستهدافها لمراقبة المرض والسيطرة عليه.

Translated from English version into Arabic by Mohamed R. Habib

基于地理探测器的安徽省血吸虫病流行环境因素评估

胡艺，夏聪聪，李石柱，Michael P. Ward，罗灿，高风华，汪奇志，张世清，张志杰

摘要

引言：血吸虫病是由裂体吸虫属血吸虫引起的介水传染病，在很多发展中国家广泛流行。这种疾病的传播通常与多种生物学特征有关，同时社会因素也对其有一定的作用。但关于各因素的交互效应对血吸虫病传播影响的研究却很少。

方法：本研究使用一系列不同的探测方法（例如：因子探测器，风险探测器，生态学探测器及交互效应探测器）评估环境因素对血吸虫病流行的影响。具体而言，(i)因子探测器通过计算基于疾病空间分布模式的决定力（Power of Determinate ,PD）指数量化风险因素对疾病的影响；(ii) 风险探测器通过空间分层探寻在某种环境因素控制下疾病的高风险区域； (iii) 生态探测器研究某环境因素对疾病空间分布模式的影响是否比其它因素更重要；(iv) 交互效应探测器比较风险

因素间的交互效应相对于各自的独立效应是削弱还是增强。血吸虫病感染数据来源于中国安徽省寄生虫病研究所的常规监测。环境因素数据来源于中国气象站和互联网上获取的国际环境数据。

结果：因子探测器确定的各因素对血吸虫病流行影响如下：长江(0.322)＞土地覆盖(0.285)＞日照时间(0.256) ＞人口密度(0.109)＞海拔(0.090)＞归一化植被指数(NDVI)(0.077)＞日间陆地表面温度(LSTday)(0.007)。风险探测器表明，血吸虫病的高危地区位于距离长江 50 千米的缓冲区内。生态学探测显示研究中的各环境因素对血吸虫病流行的影响显著不同。交互效应探测器显示，在多数情况下各因素之间的交互效应会增强它们各自的独立效应。

结论：长江对血吸虫病流行的影响作用最大，其次是土地覆盖和日照时间，其它因素影响均较弱。相比单个因素，因素间的交互效应对血吸虫病流行的影响更大。受到强交互效用影响的区域应该成为血吸虫病防控的重点区域。

Translated from English version into Chinese by Congcong Xia

Évaluation des facteurs environnementaux associés à la prévalence régionale de la schistosomiase dans la province d'Anhui, République populaire de Chine, en utilisant une méthode de détection géographique

Yi Hu, Congcong Xia, Shizhu Li, Michael P. Ward, Can Luo, Fenghua Gao, Qizhi Wang, Shiqing Zhang, Zhijie Zhang

Résumé

Contexte La schistosomiase est une maladie liée à l'eau causée par des vers trematodes appartenant au genre Schistosoma, qui prévaut dans la plupart des pays en développement. La transmission de la maladie est habituellement associée à de multiples caractéristiques biologiques et à des facteurs sociaux, qui peuvent jouer un rôle. Peu d'études ont évalué l'influence exacte de chaque facteur dans la transmission de la schistosomiase.

Méthodes. Nous avons utilisé une série de détecteurs différents (c'est-à-dire des détecteurs spécifiques, de risque, écologique et détecteur d'interaction) pour évaluer les effets individuels et interactifs des facteurs environnementaux sur la prévalence de la schistosomiase. Plus précisément, (i) un détecteur spécifique qui quantifie l'impact d'un facteur de risque sur un aspect d'observation spatiale de maladie, classés statistiquement selon une valeur calculée de puissance; (ii) un détecteur des zones à haut risque de la maladie à condition que la zone d'étude soit stratifiée selon un facteur de risque potentiel; (iii) un détecteur écologique qui explore si un facteur de risque est plus important qu'un autre dans le contrôle du modèle spatial de la maladie; (iv) un détecteur d'interaction qui explore si deux facteurs de risque pris ensemble affaiblissent ou s'améliorent mutuellement, ou s'ils sont indépendants dans le développement de la maladie. Les données sur l'infection par la schistosomiase basées sur les enquêtes classiques ont été obtenues au niveau du comté auprès des autorités sanitaires de la province d'Anhui, en Chine, et utilisées en combinaison avec des informations des stations météorologiques chinoises et des données environnementales disponibles sur le plan international.

Résultats. Le détecteur spécifique a identifié divers facteurs d'importance potentielle comme suit: la proximité avec la rivière Yangtze (0,322)> la couverture végétale (0,285)> les heures d'ensoleillement (0,256)> la densité de la population (0,109)> l'altitude (0,090)> l'indice de différence normalisée de végétation (NDVI) (0,077)> la température de la surface terrestre à la journée (LSTday) (0,007). Le

détecteur de risque a montré que les zones à haut risque de schistosomiase se trouvaient à une distance tampon de 50 km du Yangtsé. Le détecteur écologique révèle que les facteurs étudiés ont des effets significativement différents. Le détecteur d'interaction a révélé que l'interaction entre les facteurs a augmenté leurs principaux effets dans la plupart des cas.
Conclusion. La proximité de la rivière Yangtze a l'effet le plus marqué sur la prévalence de la schistosomiase, suivie de la couverture végétale et des heures d'ensoleillement, alors que les facteurs restants ont eu peu d'influence. L'interaction entre les facteurs a joué un rôle encore plus important sur la prévalence de la schistosomiase que chacun des facteurs pris séparément. Les régions à haut risque influencées par des interactions fortes doivent être ciblées pour la lutte contre la schistosomiase.

Translated from English version into French by GARBA DJIRMAY Amadou

Оценка факторов влияния окружающей среды, связанных с региональной распространенностью шистосомозом в провинции Аньхой, Китайскаая Народная Республика, используя метод географического исследования

Yi Hu, Congcong Xia, Shizhu Li, Michael P. Ward, Can Luo, Fenghua Gao, Qizhi Wang, Shiqing Zhang, Zhijie Zhang

Аннотация

Справочная информация. Шистосомоз является передаваемым через воду заболеванием, вызываемым трематодными червями, принадлежащими к роду *Schistosoma*, которые распространены в большинстве развивающихся стран. Передача заболевания обычно связана с множеством биологических особенностей и социальных факторов. Факторы могут играть важную роль. Несколько исследований смогли оценить точное влияние каждого фактора способствующего передаче шистосомоза.
Методология. Мы использовали ряд различных исследований (а именно, специфическое исследование, исследование рисков, экологическое исследование и исследование взаимодействия) для оценки отдельных и взаимодействующих влияний факторов окружающей среды на распространения шистосомоза. В частности, (i) специфическое исследование позволяет количественно оценить влияние факторов риска на наблюдаемые пространственные расположение болезни, которое ранжированы исходя из значения статистической мощности исследования; (ii) исследование рисков определяет зоны высокого риска болезни при условии, что область исследования разбита на уровни потенциальных факторов риска; (iii) экологическое исследование рассматривает, является ли один фактор сильнее другого, влияя на пространственные расположение болезни; (iv) исследование взаимодействий проверяет, усиливают или делают более слабым друг друга 2 фактора, взятые вместе или они независимы с точки зрения развития болезни. Данные по инфицированию шистосомозом, основанные на обычных обследованиях, были получены на уровне уездов/округов от органов здравоохранения провинции Аньхой, Китай и используются в сочетании с информацией от китайских метеостанций и международных данных об окружающей среде.
Результаты. Специфическое исследование выявило следующие факторы, потенциально имеющие влияние: близость к реке Янцзы (0.322) > почвенно-растительный покров (0.285) > количество солнечных часов (0.256) > плотность населения (0.109) > высота (0.090) >

нормализованный относительный индекс растительности (NDVI) (0.077) > температура поверхности земли в дневное время (LSTday) (0.007). Исследование рисков показали, что районы с высоким риском заболеваемости шистосомозом были расположены на буферном расстоянии 50 км от реки Янцзы. Экологическое исследование показало, что исследованные факторы имели существенно различное влияние. Исследование взаимодействий показало, что взаимодействие между факторами усилило их основное влияние в большинстве случаев.

Заключение. Близость к реке Янцзы имела самое сильное влияние на распространенность шистосомоза. Следующими по важности факторами были почвенно-растительный покров и количество солнечных часов, в то время как остальные факторы оказали лишь слабое влияние. Взаимодействие между факторами играет ещё более важную роль в оказании влияния на распространенность шистосомозом, чем каждый фактор в отдельности. Регионы с высоким риском, находящиеся под влиянием сильных взаимодействий, должны быть выбраны для вмешательств по борьбе с заболеванием.

Translated from English version into Russian by Dmitry Esin

Análisis de los factores ambientales asociados a la prevalencia regional de schistosomiasis en la Provincia de Anhui, República Popular de China, mediante un método de detección geográfica

Yi Hu, Congcong Xia, Shizhu Li, Michael P. Ward, Can Luo, Fenghua Gao, Qizhi Wang, Shiqing Zhang, Zhijie Zhang

RESUMEN

Antecedentes: La schistosomiasis es una enfermedad de origen acuático causada por vermes trematodos pertenecientes al género *Schistosoma*, que es prevalente en gran parte del mundo en desarrollo. La transmisión de la enfermedad está usualmente ligada a múltiples características biológicas y factores sociales que pueden jugar un papel. Son pocos los estudios que han analizado la influencia concreta de cada factor sobre la transmisión de la schistosomiasis.

Métodos: Nosotros utilizamos una serie de diferentes detectores (i.e., detector específico, detector de riesgo, detector ecológico y detector de interacción) para evaluar los efectos separados e interactivos de los factores ambientales sobre la prevalencia de schistosomiasis. Concretamente, (i) el detector específico cuantifica el impacto de un factor de riesgo sobre un patrón observado de distribución espacial de la enfermedad, que fue estadísticamente estimado por el valor de capacidad de determinación (Power of Determinate PD); (ii) el detector de riesgo detecta áreas de alto riesgo de enfermedad bajo la condición de que el área de estudio esté estratificada por un factor de riesgo potencial; (iii) el detector ecológico explora si un factor de riesgo es más significativo que otro en el control del patrón espacial de la enfermedad; (iv) el detector de interacción prueba si dos factores de riesgo cuando tomados conjuntamente debilitan o refuerzan uno al otro, o si son independientes en el desarrollo de la enfermedad. Datos de infección de schistosomiasis basados en encuestas convencionales fueron obtenidos a nivel de condado a partir de las autoridades sanitarias de la Provincia de Anhui, China, y usadas en combinación con información de estaciones meteorológicas chinas y datos ambientales disponibles internacionalmente.

Resultados: El detector específico identificó varios factores de potencial importancia tal y como sigue: Proximidad al Río Yangtze (0.322) > cubierta de campo (0.285) > horas de iluminación solar (0.256) > densidad de población (0.109) > altitud (0.090) > índice normalizado de diferente vegetación (NDVI)

(0.077) > temperatura superficial de campo durante el día (LST_{day}) (0.007).. El detector de riesgo indicó que las áreas de alto riesgo de schistosomiasis se localizaban dentro de una distancia media de 50 km del Río Yangtze. El detector ecológico dilucidó que los factores investigados tienen diferentes efectos significativos. El detector de interacción reveló que la interacción entre factores reforzaba sus más importantes efectos en muchos casos.

Conclusiones: La proximidad al Río Yangtze mostró el efecto más fuerte sobre la prevalencia de schistosomiasis seguido por la cubierta de campo y las horas de iluminación solar, mientras que los demás factores tuvieron solamente una influencia debil. La interacción entre factores jugó icluso un papel más importante en influenciar la prevalencia de schistosomiasis que cada factor por sí solo. Las regiones de alto riesgo influenciadas por fuertes interacciones necesitan ser objeto de análisis para las intervenciones de control de la enfermedad.

Translated from English version into Spanish by Màrius V. Fuentes
